# A Geospatial Assessment of Flood Vulnerability Reduction by Freshwater Wetlands–A Benefit Indicators Approach

**DOI:** 10.3389/fenvs.2019.00054

**Published:** 2019-05-03

**Authors:** Justin Bousquin, Kristen Hychka

**Affiliations:** 1 Gulf Ecology Division, National Health and Environmental Effects Laboratory, U.S. Environmental Protection Agency, Office of Research and Development, Gulf Breeze, FL, United States; 2 University of Maryland Center for Environmental Science, Cambridge, MD, United States

**Keywords:** resilience, benefit indicators, flooding, wetlands, geospatial

## Abstract

Flooding is among the most common and costly natural disasters in the United States. Flood impacts have been on the rise as flood mitigating habitats are lost, development places more people and infrastructure potentially at risk, and changing rainfall results in altered flood frequency. Across the nation, communities are recognizing the value of flood mitigating habitats and employing green infrastructure alternatives, including restoring some of those ecosystems, as a way to increase resilience. However, communities may under value green infrastructure, because they do not recognize the current benefits of risk reduction they receive from existing ecosystems or the potential benefits they could receive through restoration. Freshwater wetlands have long been recognized as one of the ecosystems that can reduce flood damages by attenuating surface water. Small-scale community studies can capture the flood-reduction benefits from existing or potentially restored wetlands. However, scalability and transferability are limits for these high resolution and data intensive studies. This paper details the development of a nationally consistent dataset and a set of high-resolution indicators characterizing where people benefit from reduced flood risk through existing wetlands. We demonstrate how this dataset can be used at different scales (regional or local) to rapidly assess flood-reduction benefits. At a local scale we use other national scale indicators (CRSI, SoVI) to gauge community resilience and recoverability to choose Harris County, Texas as our focus. Analysis of the Gulf Coast region and Harris County, Texas identifies communities with both wetland restoration potential and the greatest flood-prone population that could benefit from that restoration. We show how maps of these indicators can be used to set wetland protection and restoration priorities.

## INTRODUCTION

Flooding is a significant problem across the United States, both in terms of contribution to overall risk (Doocy et al., 2013; Summers et al., 2017) and recovery costs (ASFPM, 2013). Four confounding trends are increasing the impact of flooding: changing occurrence and intensity of storm events (Milly et al., 2008), increased urbanization (Hollis, 1975; Shuster et al., 2005), increased value of assets vulnerable to flooding (Jongman et al., 2012), and decreased stormwater mitigation due to loss of green infrastructure (Ahern, 2007).

Green infrastructure refers to the systems that provide services to people—these systems can be managed to varying degrees from fully constructed systems, such as rain gardens, to existing, “natural” ecosystems, like floodplain wetlands (Burgess, 2015). The ability of wetlands to function by attenuating flood waters depends primarily on their type, condition, and landscape position (Brinson, 1993; Bullock and Acreman, 2003; Acreman and Holden, 2013; Kadykalo and Findlay, 2016). When a wetland attenuates flood water it has the potential to provide an ecosystem service, in the form of flood-reduction services. Despite the critical role wetlands play in the landscape, their extent in the contiguous United States has declined by more than half since colonization (Dahl, 1990). Most freshwater wetland losses are due to historic drainage to convert those areas to productive agricultural lands (Dahl and Allord, 1996) and continuing urban development (Stein and Ambrose, 1998; Kelly, 2001).

Recognizing both the magnitude of wetland losses, and the resulting loss in the vital ecosystem services those wetland systems supplied, national regulatory policies and community grassroots actions have focused on protecting remaining wetlands and restoring wetlands where feasible (Dahl and Allord, 1996). Federal programs include the Federal Emergency Management Agency (FEMA) Community Rating System, an incentive-based program that credits preservation or restoration of wetlands in floodplains, and the Department of Agriculture’s Conservation Reserve Program, which funds wetland restoration projects nationally (USDA, 2015; FEMA, 2017). Community organizations restore, protect, and otherwise manage wetland resources through a variety of mechanisms, including mitigation banks, acquisitions, and easements (Environmental Law Institute, 2012).

With numerous protection or restoration candidate sites and limited funding, practitioners have developed many strategies to prioritize sites for restoration. Several states have developed their own tools or assessments, such as Rhode Island’s adaptation of a rapid assessment tool (Miller and Golet, 2001), Oregon’s Wetland Restoration Planning Tools^1^, or Wisconsin’s Wetlands and Watersheds Explorer (Miller et al., 2017). Most existing wetland assessment methods focus on wetland functions. Where methods do address benefits, it is typically in the form of a judgment regarding the “social significance” of each function (see King et al., 2000 and King and Price, 2004 for more on these approaches). Healy and Secchi (2016) reviewed seven ecosystem service modeling and decision support tools (e.g., Invest; Nelson et al., 2009) for their ability to evaluate different wetlands restoration scenarios. Only the ARIES model (Bagstad et al., 2014), incorporated benefits of inland flood-reduction services. However, the approach required semi-intensive modeling making it difficult to apply as a screening tool or at broader scales. Economic valuation studies are another approach that evaluate tradeoffs between alternative restoration sites by placing dollar values on wetlands or specific services of wetlands (Brander et al., 2006, 2013; Ghermandi et al., 2010). However, transferring the results of previous studies quickly and consistently across national or regional scales is difficult, because values are context and location-specific, requiring additional information for benefit transfer and use in local decision making.

The Rapid Benefit Indicators (RBI) approach (Mazzotta et al., 2016, 2018) attempts to directly capture the potential benefits to people from wetland restoration or protection efforts. It outlines an indicator set for prioritizing wetlands within a watershed informed by how people interact with, benefit from, and value the wetlands.

This paper details how we assembled a national dataset of spatial indicators developed through the RBI approach. We demonstrate how to use the developed indicators to inform decisions on wetland restoration and conservation priorities at multiple scales. Locations where there is demand for flood-reduction services by populations in potentially flooded areas downstream but there is limited supply of those services from wetlands upstream indicates restoration priorities. Locations where people currently benefit from reduced flooding through existing wetlands indicate conservation priorities.

## METHODS

The Rapid Benefit Indicators (RBI) approach is a beneficiary-centric, non-monetary way to quickly compare restoration sites using indicators that address several questions related to the aspects of benefits that make them valuable. Spatial tools developed to apply the RBI approach answer these questions by assessing indicators for the area surrounding potential restoration sites from spatial datasets (Bousquin et al., 2017). Most of the spatial input datasets have suggested national defaults for use where locally relevant data are not available. We developed a new national dataset as a more accessible alternative to the spatial analysis tools. The dataset has the added benefit that practitioners can use it at multiple scales. The new dataset is specific to flood-reduction services and answers two of the RBI questions.

The first question addressed here is: how many people benefit? In contrast to some other benefits of freshwater wetlands, flood-reduction is particularly dependent on the spatial and hydrological characteristics of the landscape, because sites providing flood-reduction services benefit people some distance downstream. The value of flood-reduction services is highly dependent on the number of people who benefit as well as the value and level of vulnerability of assets protected from flooding. During a storm event people benefit from flood-reduction services in the form of flood damage reductions. When planning for future storms, the benefit of future flood-reduction services can take the form of flood-risk reductions. Here, we use the term flood-reduction benefits to refer to both benefits interchangeably. The number of people downstream who are currently exposed to flooding and may experience flood-reduction benefits after site restoration indicates how many people benefit. For simplicity this will be referred to as the demand indicator.

The other question addressed here is: by how much do people benefit? This question has four parts, however the only one addressed here is service scarcity. For flood-reduction services, people downstream may already be receiving benefits from existing wetlands or gray infrastructure, such as dams and levees. Where there are fewer substitute sources of flood-reduction services, those services are scarcer and the value of flood-reduction services from a restored site is greater. The prevalence of wetlands upstream provides an indication of the existing flow of benefits. For simplicity this will be referred to as the scarcity indicator.

The national dataset we developed characterizes two metrics: the population in flood-prone areas and the prevalence of existing wetlands. However, the spatial relationship of a restoration site, either upstream or downstream of these landscape characterizations is what identifies the beneficiaries or service scarcity. We used the perspective of a practitioner evaluating a potential restoration site to frame the spatial relationship. From that perspective the indicators are (1) downstream beneficiaries, i.e., how many people are in flood-prone areas that would benefit from restoring the site, and (2) local scarcity, i.e., how many wetlands are already supplying the same flood-reduction services. The alternative perspective is that of a potential beneficiary and could be explored using the same national dataset and methods (see Supplemental Materials). From that perspective the indicators would be (1) local beneficiaries and (2) upstream scarcity.

Another requirement for this national dataset was scalability. When a county, municipality, watershed manager, or other community decision maker is trying to prioritize wetlands restoration or conservation projects on the ground, the data used to inform that decision needs to be high resolution and aggregated to a scale appropriate to discern differences within that management area. Both regional- and local-scale decision makers need to be able to use the dataset to prioritize restoration or conservation. We developed the national dataset for use at a local-scale but demonstrate a method for aggregating information for use in regional prioritization.

### Case Study Selection

Other resilience indicators and characterizations may lend important context to restoration and conservation decisions. Often this type of information follows sociopolitical jurisdictions instead of natural boundaries. We used the Climate Resilience Screening Index (CRSI; Summers et al., 2017) to identify Harris County, TX as an area that might benefit from green infrastructure. The CRSI uses scores to characterize county resilience to a variety of natural risks. Five domains comprise the index, each including a set of indicators and metrics. Summers et al. (2018) details CRSI component domain scores, each scaled 0.01–0.99 for the nation. These domain scores provide additional insight to other county resilience factors. Risk is the first domain (0.99 for Harris County), where a higher Risk score means greater risk of hazardous events occurring. Harris County includes the City of Houston and surrounding areas, meaning there may be added redundancy and resilience infrastructure that comes with a major city, reflected in a high score for the built environment domain (0.84) that offsets some risk. The natural environment domain is of special interest here, as it includes indicators describing the extent and condition of natural ecosystems in the context of natural hazards resilience. A low natural environment domain, 0.19, demonstrates Harris County has limited resilience from natural systems. A county with a low score in the CRSI natural environment domain has the opportunity to increase their resilience through green infrastructure or restoration.

Another natural hazards resilience characterization, the Social Vulnerability Index (SoVI; Cutter et al., 2003), utilizes census demographic data, often available at the census tract level, to characterize socio-economically vulnerable populations. We used SoVI to identify beneficiaries that are the most socio-economically vulnerable and may need resilience most because they have fewer resources to recover from natural hazards. Census tract SoVI scores within Harris County range from −6.93 to 11.85, when scaled within Texas (Hazards Vulnerability Research Institute, 2014). The average SoVI score for the 786 tracts in Harris county is −0.237, considerably less than the Texas average (11.85), indicating vulnerable populations in the county.

Both resilience indicators, CRSI and SoVI, confirmed Harris County Texas as a good candidate for the local scale demonstration. We chose the Gulf of Mexico for our regional demonstration because Harris County falls within that region and because other manuscripts within this special issue also highlight other indicators in the Gulf of Mexico.

### Framework

The existing hydrologic framework of the Nation’s rivers and streams (National Hydrography Dataset Plus Version 2; McKay et al., 2012) provides a way to connect upstream and downstream areas. Several approaches have used this framework to connect upstream landscape characteristics (Hill et al., 2016) or contaminant sources (Samuels et al., 2014) with downstream conditions. Here the framework connected upstream flood attenuating wetlands to people that benefit in downstream flood-prone areas.

NHDPlusV2 defines catchments for the area that flows into each stream reach, hereafter referred to as “catchments.” These small-scale catchments, at an average size of 3.1 km^2^, are at a useful scale for localized decisions. NHDPlusV2 integrated the latest Watershed Boundary Dataset (WBD), allowing catchments to be cross-walked to eight-digit WBD Hydrologic Unit Codes (HUC-8). The integration and crosswalk to HUC-8 (average size of 3,805 km^2^) allows for aggregation to broader scales for use in regional decisions.

### Characterizing Catchments

**FIGURE 1 |:**
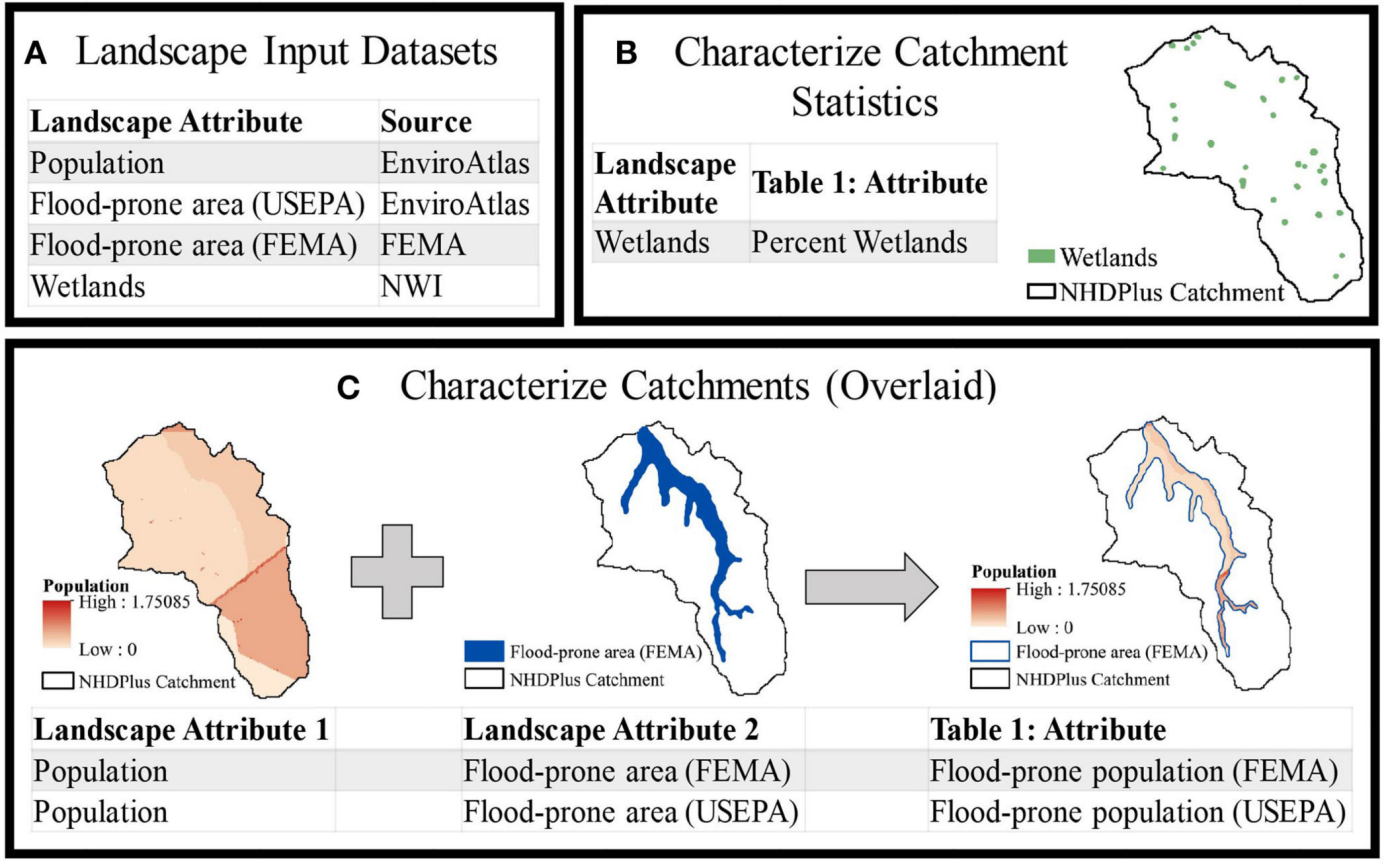
Workflow used to characterize catchment attributes using **(A)** landscape input datasets. Catchments were characterized by landscape attributes either **(B)** as a percent of catchment area, for wetlands, or **(C)** as a total after being overlaid with a flood-prone-area, for flood-prone population.

Each catchment required two new attributes: wetland extent and flood-prone population. Figure 1 lists the four main input datasets (Figure 1A) and how we used them to characterize the catchment attributes listed in Table 1.

We characterized wetlands as a percent of each catchment (Figure 1B) using the National Wetland Inventory (NWI; USFW, 2018). The NWI is a national characterization of wetland extent and type. Of the defined types, we limited wetlands in this study to “Freshwater Emergent Wetland,” “Freshwater Forested/Shrub Wetland,” and “Freshwater Pond.” We excluded non-freshwater coastal wetlands, because they provide flood-reduction services by reducing storm surge energy, acting as a barrier to water coming inland from the coast rather than by attenuating flood waters. An advantage of NWI over the National Land Cover Database (NLCD; Homer et al., 2015) is that the NWI differentiates more wetland types and is a more accurate representation of wetland extent in at least some settings (Handley and Wells, 2009).

Determining the flood-prone population required spatial information on flood-prone areas and population. We used a dasymetric raster representation of block-level 2010 census populations available for the contiguous United States through the USEPA’s EnviroAtlas (Pickard et al., 2015; USEPA, 2016). This dasymetric mapping redistributes populations within census block polygons based on land use and slope to reassign population from areas within the census polygons where people are unlikely to live to where the population is likely denser (USEPA, 2015). Re-distributing populations within census blocks is particularly important for flood analysis (Merz et al., 2010), where census block geometries frequently overlap waterbodies (lakes, rivers etc.) leading to flood-prone population overestimation. To identify beneficiaries, the population input dataset was overlaid with a flood-prone area (Figure 1C). We used two datasets to delineate the flood-prone area, FEMA (FEMA, 2018) and USEPA (Woznicki et al., 2019).

FEMA maps flood zones under the National Flood Insurance Program (NFIP). NFIP uses flood zones for insurance purposes, and the maps do not necessarily reflect observed flooding. The FEMA flood maps consider conditions at the time of creation, including landscape attenuation and any gray infrastructure such as dams or levees. FEMA characterizes flood zones based on recurrence (e.g., 100-year and 500-year storms) and based on cause (riverine is an A-zone, coastal is a V-zone). FEMA makes flood zone data available in a variety of ways. The Federal Insurance and Mitigation Administration (FIMA) national vector dataset (FEMA, 2018) best fit the needs of this study. We restricted the flood zones considered to 100-year A flood zones (A, AE, AO, AH) and combined adjacent flood zone polygons to remove duplicates and speed up processing.

FEMA flood maps have drawbacks. FEMA designs flood maps with the intent to revise and update them over time, but revision costs can be inhibitive resulting in many outdated flood maps. Additionally, FEMA flood maps are not comprehensive, leaving large areas of the United States unmapped.

Several modeling efforts have attempted to fill gaps in the FEMA flood maps by creating new nationally consistent maps (Wing et al., 2018; Woznicki et al., 2019). Woznicki et al. (2019) did this by training probabilistic models on ancillary data where 100-year FEMA maps were available and then applying those models to predict flood-prone areas from the same ancillary data in unmapped regions. The models performed well, accurately classifying 79% of the FEMA flood zones. FEMA maps are more versatile, because they differentiate types of flooding, and are more accurate, incorporating local data and gray infrastructure into their modeling. However, for 40% of contiguous United States there are no FEMA maps, making the dataset hosted by EnviroAtlas and described in Woznicki et al. (2019) a more comprehensively available alternative. Because both input datasets are useful in different parts of the contiguous United States depending on availability of FEMA data, we used both to characterize flood-prone areas of catchments.

We adapted scripts, algorithms, and quality assurance procedures from those used to characterize catchments and produce watershed summaries for the StreamCat dataset (Hill et al., 2016). Whereas, StreamCat converted vector landscape features to raster before catchment characterization, here we characterized catchments using the original vector or raster data. Adapted python scripts are available in Supplemental Materials. Table 1 lists the fields used to characterize catchments in the new national dataset we developed.

### Using Networked Catchments for Upstream/Downstream and HUC-8 Aggregate Metrics

**FIGURE 2 |:**
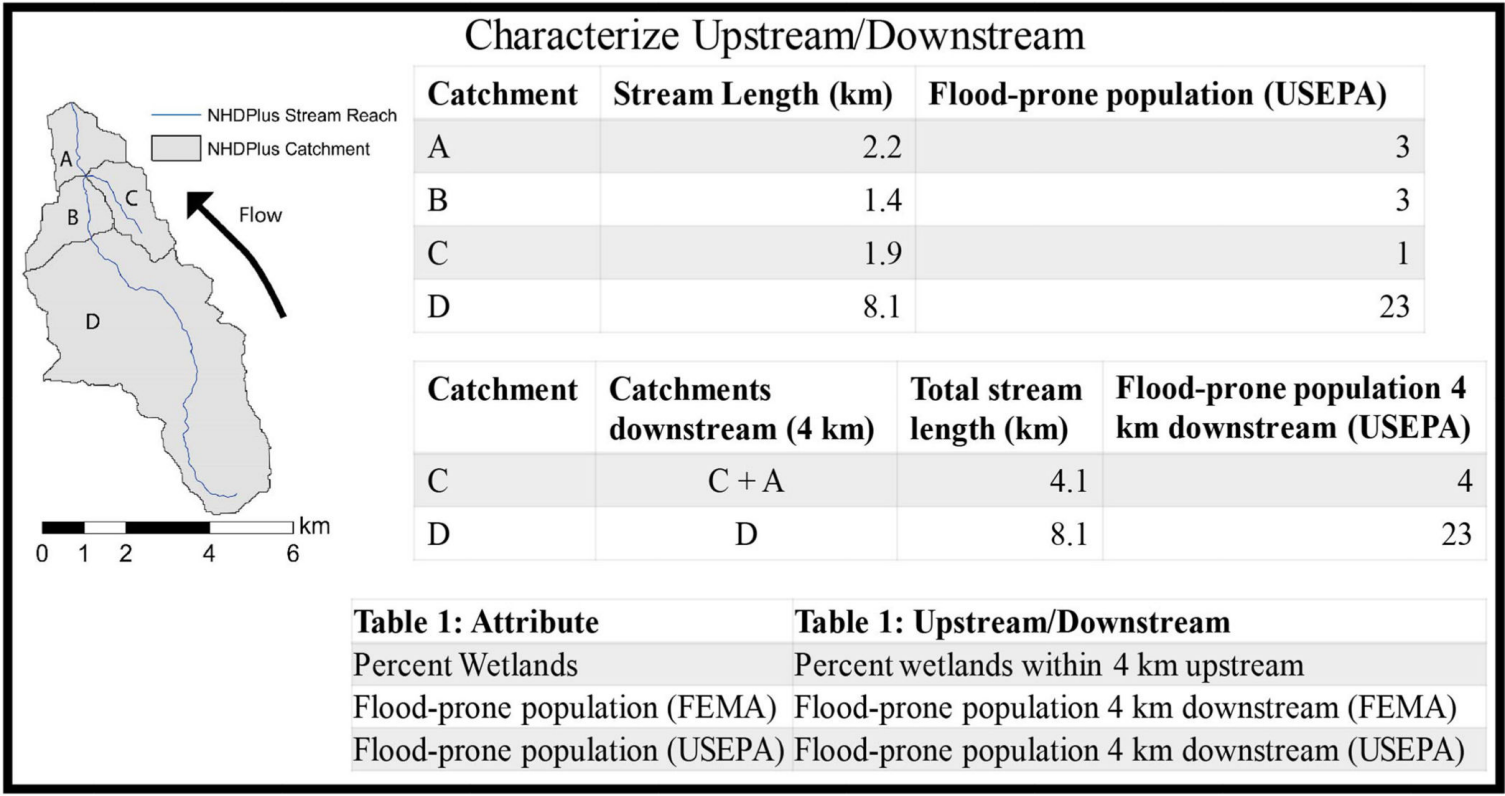
Example of how upstream/downstream catchment metrics were calculated from catchment attributes.

With catchments characterized, the networked feature of those catchments allows for the calculation of upstream and downstream metrics. Figure 2 shows how we used catchment attributes (Figure 1B) and a specified stream length to calculate the “Upstream/Downstream” metrics (Table 1).

Understanding the sensitivity of results to the specified upstream or downstream stream length limit is vital since the distance flood-reduction benefits travel downstream may be different for different areas, different wetlands, or even for the same wetland during different storms (Mitsch and Gosselink, 2000; VanSickle and Burch-Johnson, 2008; Liu et al., 2014; Bousquin et al., 2015). We explored the sensitivity of three stream length limits (4-km, 5-km and 8-km; see Supplemental Materials) chosen based on simulations of wetland restoration (Bousquin et al., 2015). A catchment upstream/downstream total included a downstream catchment if the total stream length from that downstream catchment to the catchment was less than the stream length limit (i.e., 4 km). Figure 2 demonstrates calculation of flood-prone population 4 km downstream for two example catchments, C and D. For catchment C in Figure 2 the flood-prone population downstream includes the flood-prone population from catchments C and A. The total stream length for these two catchments is 4.1, so no catchments further downstream are considered. If a catchment contains a stream length greater than the stream length limit, e.g., catchment D in Figure 2, the catchment upstream/downstream characterization will only represent characteristics in that catchment. If there are no upstream catchments (e.g., headwater catchments) or no downstream catchments (e.g., terminal catchments or a sink catchment) the catchment upstream/downstream characterization may only represent characteristics in that catchment.

We combined catchment data by HUC-8 using the WBD snapshot data included with NHDPlusV2 for regional analysis. We either totaled metrics in component catchments (e.g., FEMA based flood-prone population) or calculated the area-weighted percent of the catchments (e.g., NWI based wetlands percent). Sink catchments do not contribute to downstream flows and are not included in the HUC-8 data.

### Assigning Catchment Priorities

We demonstrate how the scarcity and demand indicators can be used to assign regional and local priorities. At the regional scale, we selected the sub-regions that drain into the Gulf of Mexico for demonstration and aggregated metrics to prioritize by HUC-8. At the local scale, we selected Harris County, Texas for demonstration and assigned priorities at the catchment level. We selected Harris County, TX because it is a good candidate for ecosystem restoration based on CRSI and SoVI, and because it has experienced recent flood events.

We assigned each HUC-8 or catchment a priority based on a quartile distribution for that indicator. For the Gulf of Mexico, the quartiles were from values within the region (329 HUC-8s). For the local comparison, the quartiles were from values in the NHDPlusV2 sub-region, in this case the 24,607 catchments in the Texas 12a sub-region.

We chose quartiles over more quintiles for two reasons. First, the high number of catchments with no wetlands or no flood-prone areas caused smaller bins to be redundant (e.g., in some cases 40% of catchments might have no wetlands). Second, in the example application, we placed catchments into 3 main categories, limiting the utility of increased levels of discretization. Decision makers should choose binning methods and categories that best meet their needs.

### Quality Assurance and Missing Data

Input datasets stopped at international borders or contained missing data in some locations. Where possible, we estimated the percent of each catchment that was missing values for each input dataset (see Supplemental Materials). Neither the NWI or the FEMA flood-prone area datasets identify areas of missing values and catchments do not have percent missing for these data. For FEMA flood zones, we encourage users to evaluate missing values using FIRM boundaries^2^.

## RESULTS

This study presents results from national, regional, and local analysis. At the national scale, results focus on catchment characterizations. At the regional scale, results focus on the same characterizations but aggregated to HUC-8 for the Gulf of Mexico. At the local scale, results include catchment landscape characterizations, upstream/downstream characterizations, and quartile designations. We use an example application in Harris County, Texas to demonstrate how practitioners could use local-scale data for restoration and conservation decisions.

### National Results

We characterized catchments for use in benefit analysis in two ways: the percent of wetlands and the number of people living in flood-prone areas within the catchments themselves. Results for all catchment characterizations listed in Table 1 are available as comma separated values in the Supplemental Materials.

**FIGURE 3 |:**
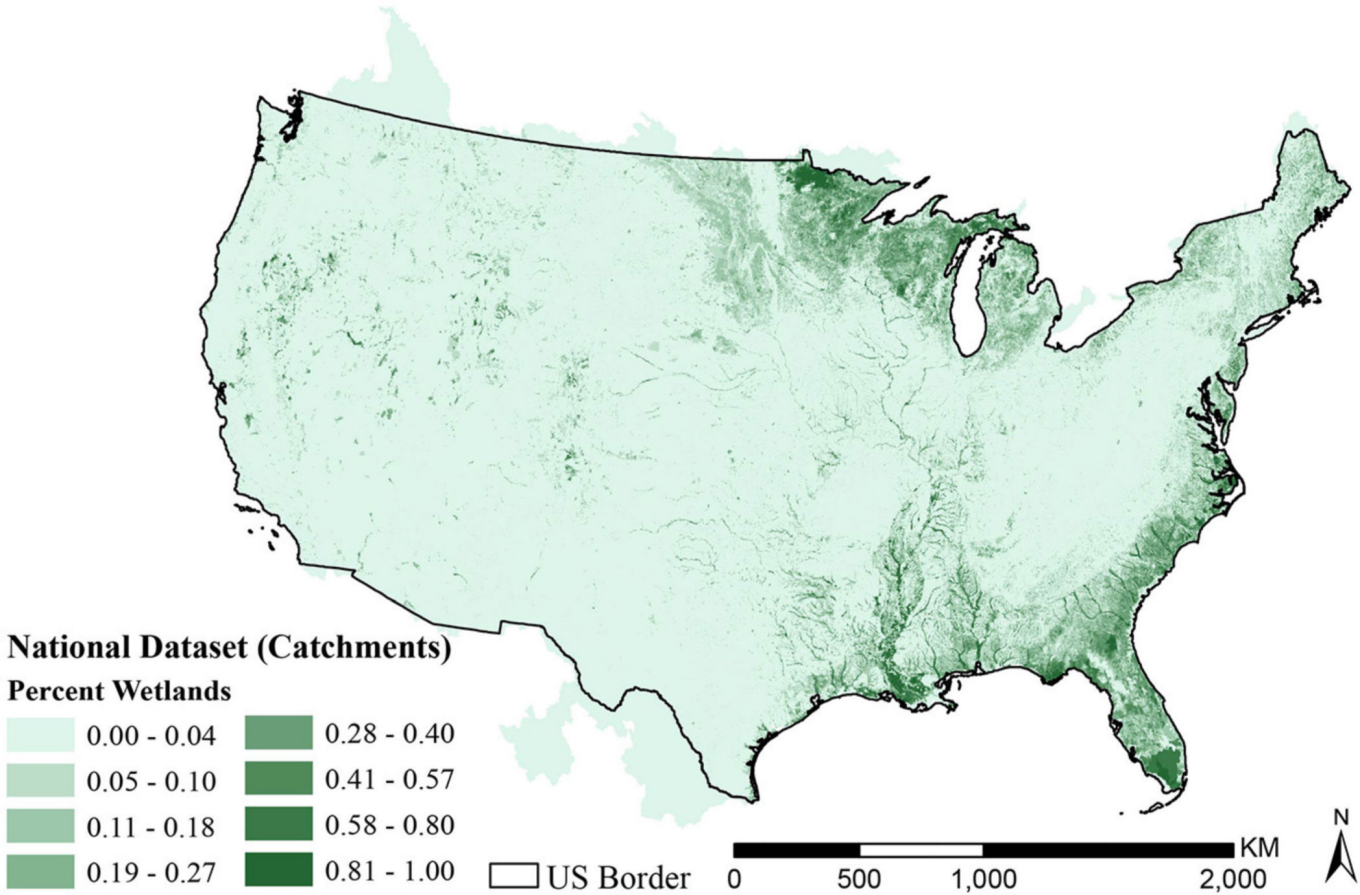
Map of catchment percent wetlands in the contiguous United States. Catchment values range from 0.00 to 1.00. The US border is used to show where the NWI dataset stops, indicating catchments outside that border include areas of missing data.

The national map of catchment percent wetlands in Figure 3 shows physiographic trends in freshwater wetlands. There is higher percent wetlands cover in the Great Lakes, along the Mississippi, and along the East Coast. These catchment characterizations for wetlands compared favorably to characterizations StreamCat made using other wetland datasets (see Supplemental Materials).

**FIGURE 4 |:**
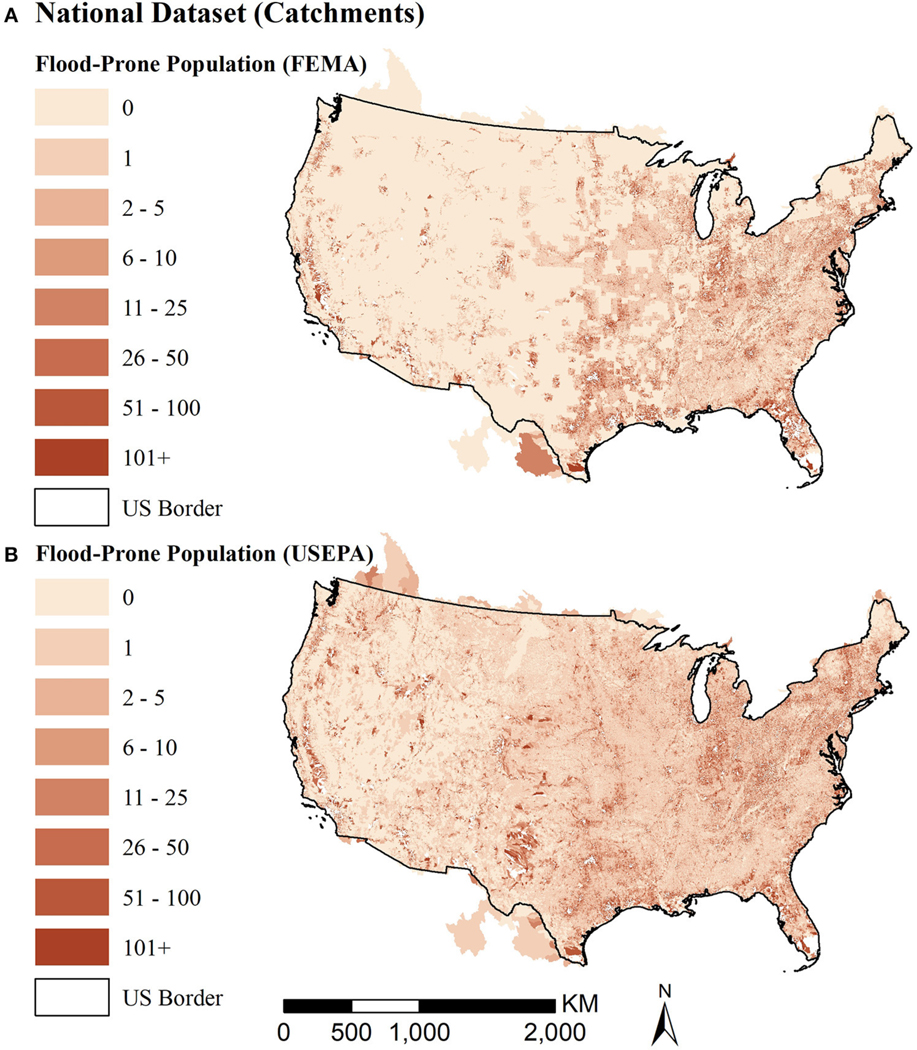
Maps of population in flood-prone areas using FEMA **(A)** and EnviroAtlas **(B)** flood zone extents. Colors for the number of people in flood prone areas within each catchment use a consistent scale across the two maps to facilitate comparisons.

The two national maps in Figure 4 show the total dasymetric population in flood-prone areas: the first using the FEMA flood zones and the second using USEPA modeled flooding. Both maps show flooding occurs throughout the contiguous United States. The map showing population in the USEPA flood-prone areas detects more widespread flood-prone population west of the Mississippi than the map showing population in the FEMA flood zones, likely due to FEMA non-mapped areas. Values in catchments that extend outside of US borders likely include areas with missing values in both flood-prone area definitions.

### Gulf Region

National maps show trends in catchment-level wetland and flood-prone population. Decisions based on the combination of this information are best made at regional- and local-scales to avoid weighing national physiographic trends too heavily. This section explores how regional decision makers could use these results in the Gulf of Mexico.

**FIGURE 5 |:**
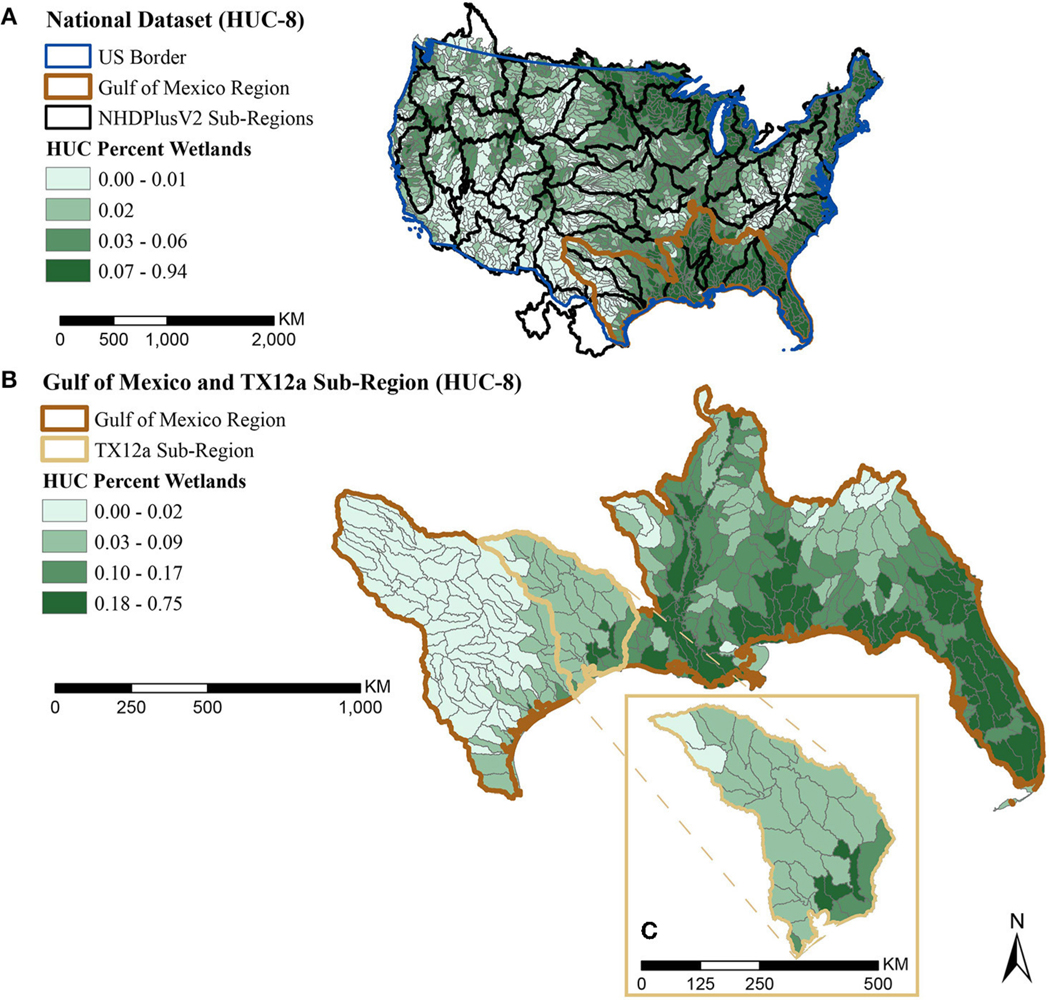
Map of HUC-8 percent wetland quartiles **(A)** scaled nationally, with the Gulf of Mexico region highlighted in brown **(B)** scaled to the Gulf of Mexico region with the TX 12a sub-region highlighted in tan, and **(C)** an expanded highlight showing the HUC-8 watersheds within the TX 12a sub-region.

**FIGURE 6 |:**
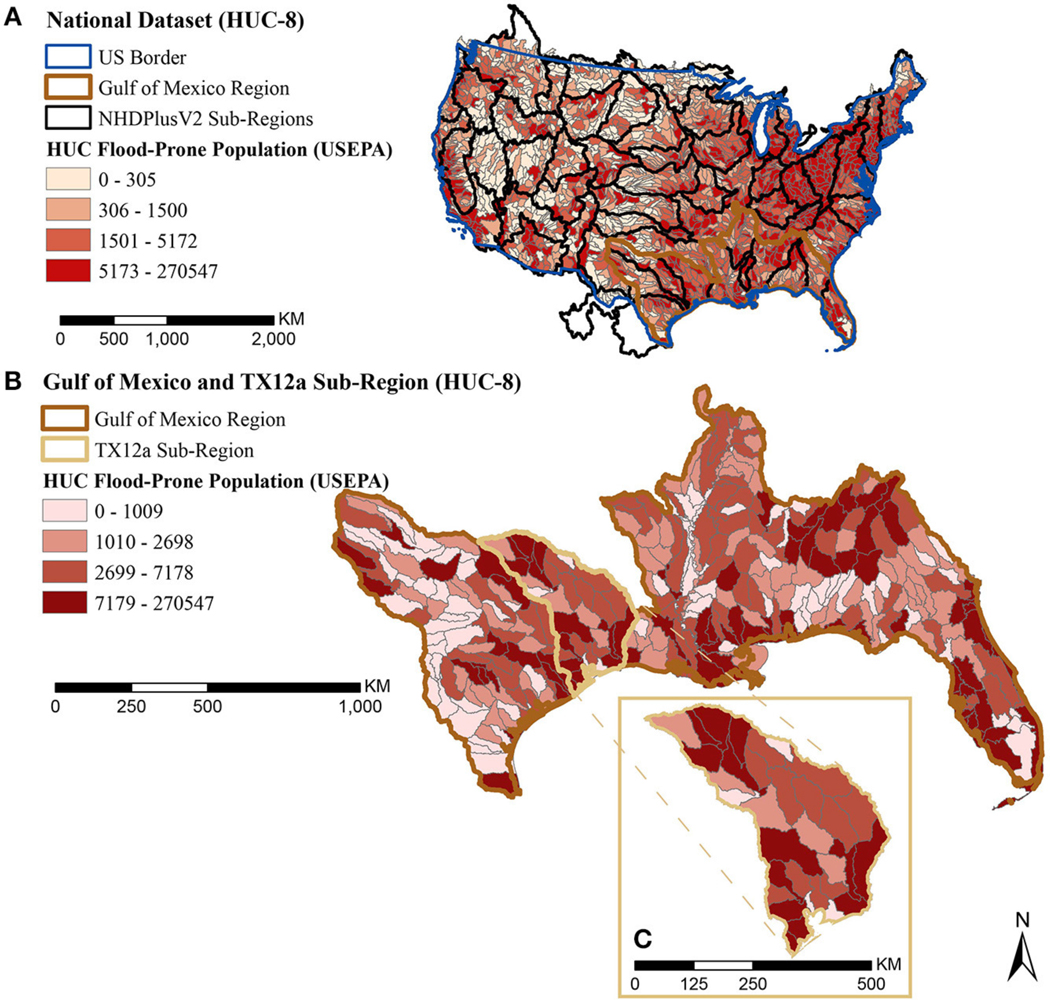
Map of HUC-8 population in USEPA flood-prone area quartiles **(A)** scaled nationally, with the Gulf of Mexico region highlighted in brown **(B)** scaled to the Gulf of Mexico region with the TX 12a sub-region highlighted in tan, and **(C)** an expanded highlight showing the HUC-8 watersheds within the TX 12a sub-region.

We aggregated catchment characterizations up to HUC-8 for regional results. Figures 5, 6 show HUC-8 results for the same two characterizations from national catchments (Figures 3, 4): percent wetlands based on NWI (Figure 5) and total population in USEPA flood-prone areas (Figure 6). We choose to use USEPA flood-prone areas for regional-scale decisions, because aggregation to HUC-8 generalizes data rich areas with areas missing data making it harder to detect missing data in the FEMA dataset. The Harris County results section revisits the FEMA flood maps at the local-scale decision level where catchments with missing values are easier to identify.

The HUC-8 map of wetlands shown in Figure 5 suggests some differences in physiography across the region, with western Texas showing limited existing wetlands. This contrasts the abundance of wetlands along the Mississippi and on the Florida peninsula. Areas in the second quartile for percent wetlands may be ideal for restoration, since the presence of some wetlands suggests the area is suitable, but the percent wetlands may not be high enough to significantly reduce flood waters in the watershed (3–7 percent of watershed suggested by Hey and Philippi, 1995).

Figure 6 shows HUC-8 total flood-prone population. Greater demand for flood-reduction benefits will be in areas where there is a greater population in flood-prone areas, areas in the 4th quartile. The proximity of wetlands to the population in flood-prone areas was not explored at this scale.

Ideal areas for further investigation are those with a high demand, indicated by quartile 4 for the number of people in flood-prone areas, and moderate scarcity, indicated by the second quartile for percent wetlands.

### Harris County, Texas

At a local-scale we focus on Harris County, Texas. Based on CRSI generalized resilience and its component domains, Harris County has an opportunity for increased resilience through green infrastructure. We show how a county can use a benefits approach to inform green infrastructure decisions, such as wetlands restoration.

#### Catchment Characterization

Our focus is on two catchment characterizations for use as indicators: catchment percent wetlands, indicating scarcity, and flood-prone population 4 km downstream, indicating demand. Characterizing the flood-prone population 4 km downstream involved four other catchment characterizations: percent flood-prone area (USEPA), percent flood-prone area (FEMA), total population and flood-prone population (USEPA).

**FIGURE 7 |:**
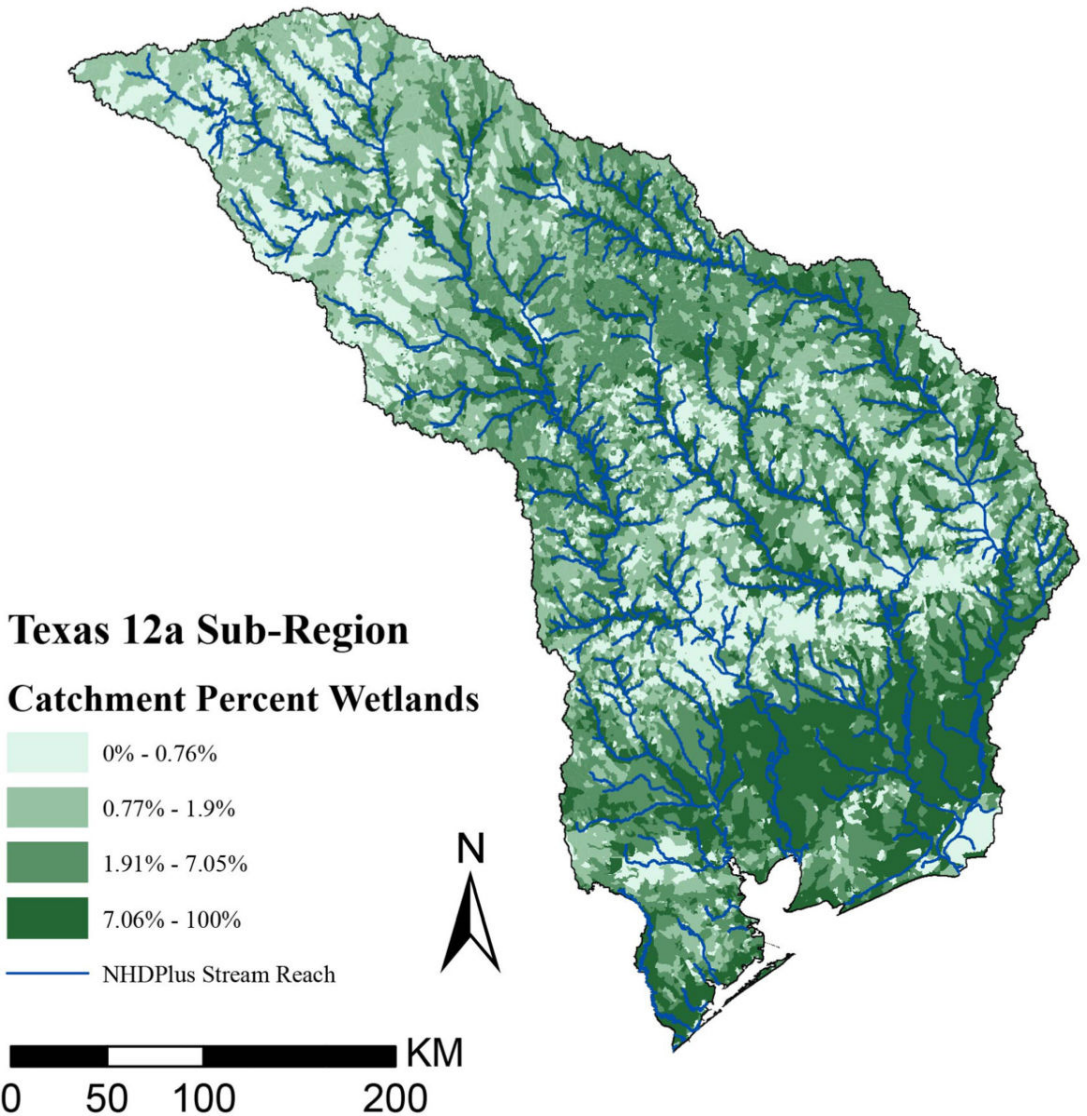
Map of Texas 12a sub-region catchment percent in NWI wetlands quartile. This map also shows NHDPlus reaches with a stream order greater than two in blue.

The map in Figure 7 shows catchment percent wetlands classified into quartiles for the 12a sub-region. We use this characterization of wetlands as a scarcity indicator. Catchments with more wetlands are near main stream and river flow paths rather than in headwaters. Wetlands are also more prevalent near the southern coast.

**FIGURE 8 |:**
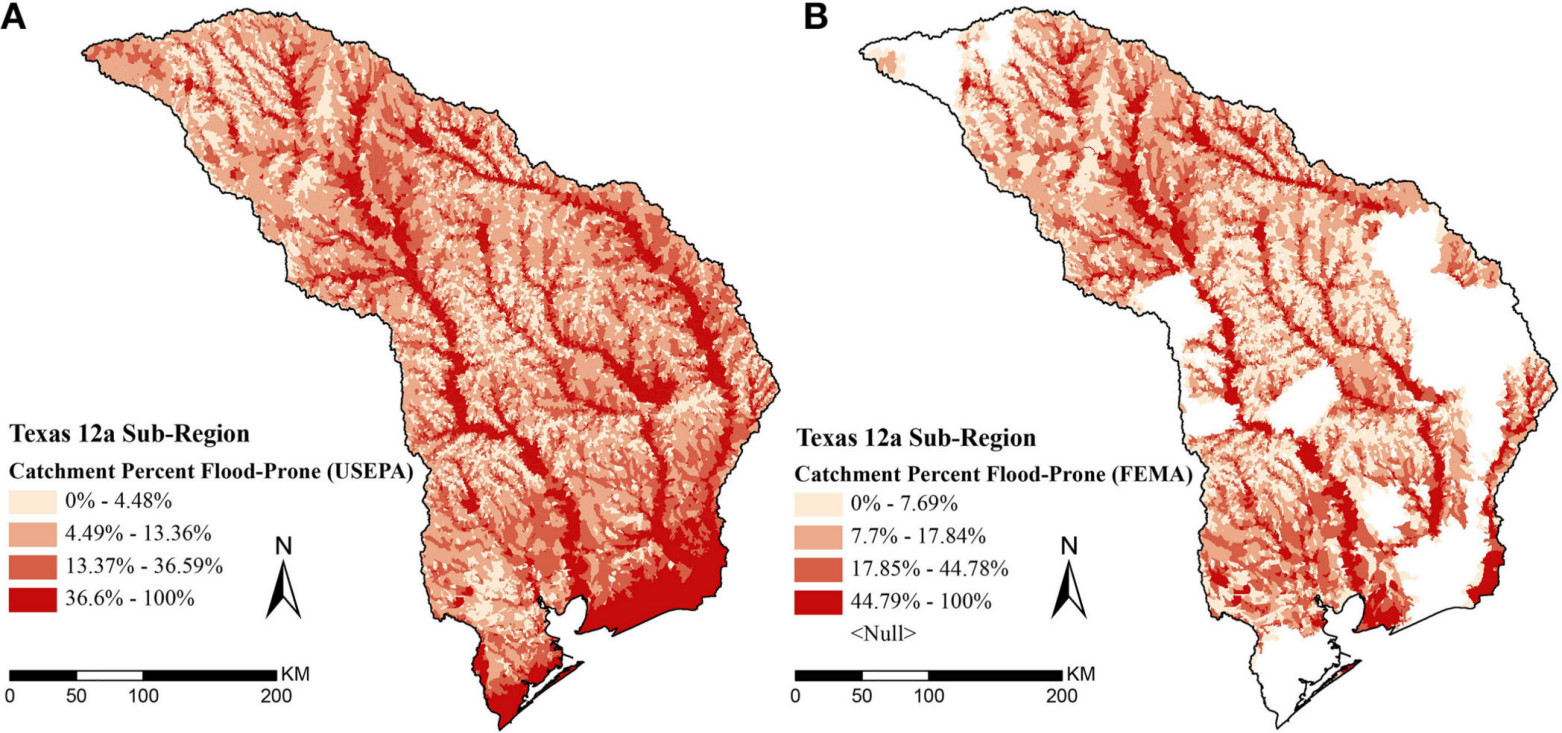
Map of Texas 12a sub-region catchment percent flood-prone area quartiles **(A)** using USEPA flood model, or **(B)** using FEMA A flood zones.

The maps in Figure 8 show catchment percent flood-prone, using either USEPA flood-prone area (Figure 8A) or FEMA flood zones (Figure 8B) to highlight differences between the two datasets and demonstrate how to choose one for use in local benefit assessment. Catchments lacking FEMA flood zones are apparent as large white spots on the map where data is missing. These gaps are largely due to un-mapped areas, but removal of vector zones may contribute to missing data in coastal areas. We chose the USEPA dataset to map flood-prone populations in Harris County, because of gaps in FEMA coverage along the southern part of Harris County, an area that also contains high population. It is worth noting that FEMA is currently updating flood maps in this area. In other parts of the same map, e.g. southern Dallas, FEMA better accounts for flooding making it a better choice for use in decision making there.

**FIGURE 9 |:**
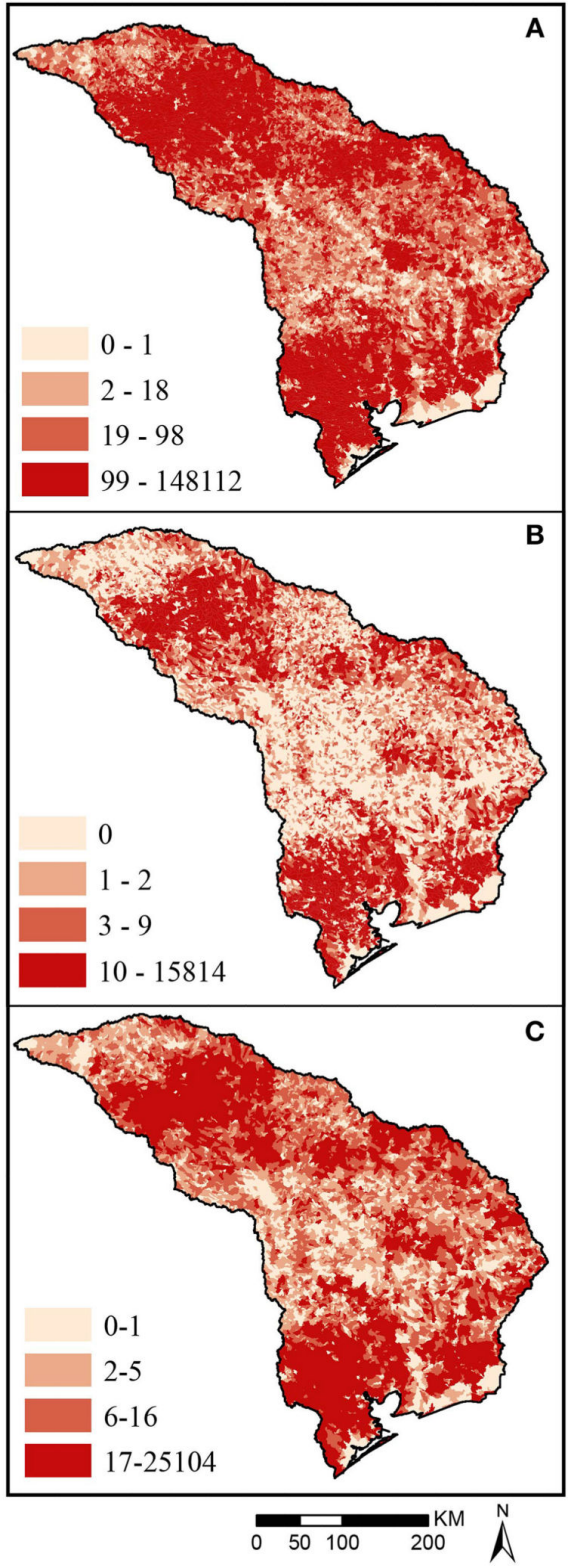
Map of Texas 12a sub-region catchment quartiles for **(A)** population **(B)** flood-prone population using USEPA flood model, and **(C)** flood-prone population within 4 km downstream. Each map uses colors based on quartiles of values for all catchments shown on the map.

The catchment population (Figure 9A) is most concentrated in the south, near Houston, and in the north, near Dallas. As described in Characterizing Catchments and Figure 1C, the catchment population (Figure 9A) was overlaid with USEPA flood-prone areas (Figure 8A) to characterize each catchment by the population in flood-prone areas (Figure 9B). As described in Using Networked Catchments for Upstream/Downstream and HUC-8 Aggregate Metrics, and Figure 2, we combined the catchment population in flood-prone areas (Figure 9B) for catchments within 4 km downstream (Figure 9C), characterizing catchment flood-prone population 4 km downstream (USEPA) for use as a demand indicator.

Upper quartile limits in Figure 9 legends may be misleading, as the same, outlier catchment has both the highest flood-prone population (Figure 9B) and the highest downstream flood-prone population (Figure 9C), 15,814 and 25,104, respectively. The next highest catchment for both metrics is significantly lower (9,289 and 9,340 respectively).

#### Benefit Assessment and Prioritization

**FIGURE 10 |:**
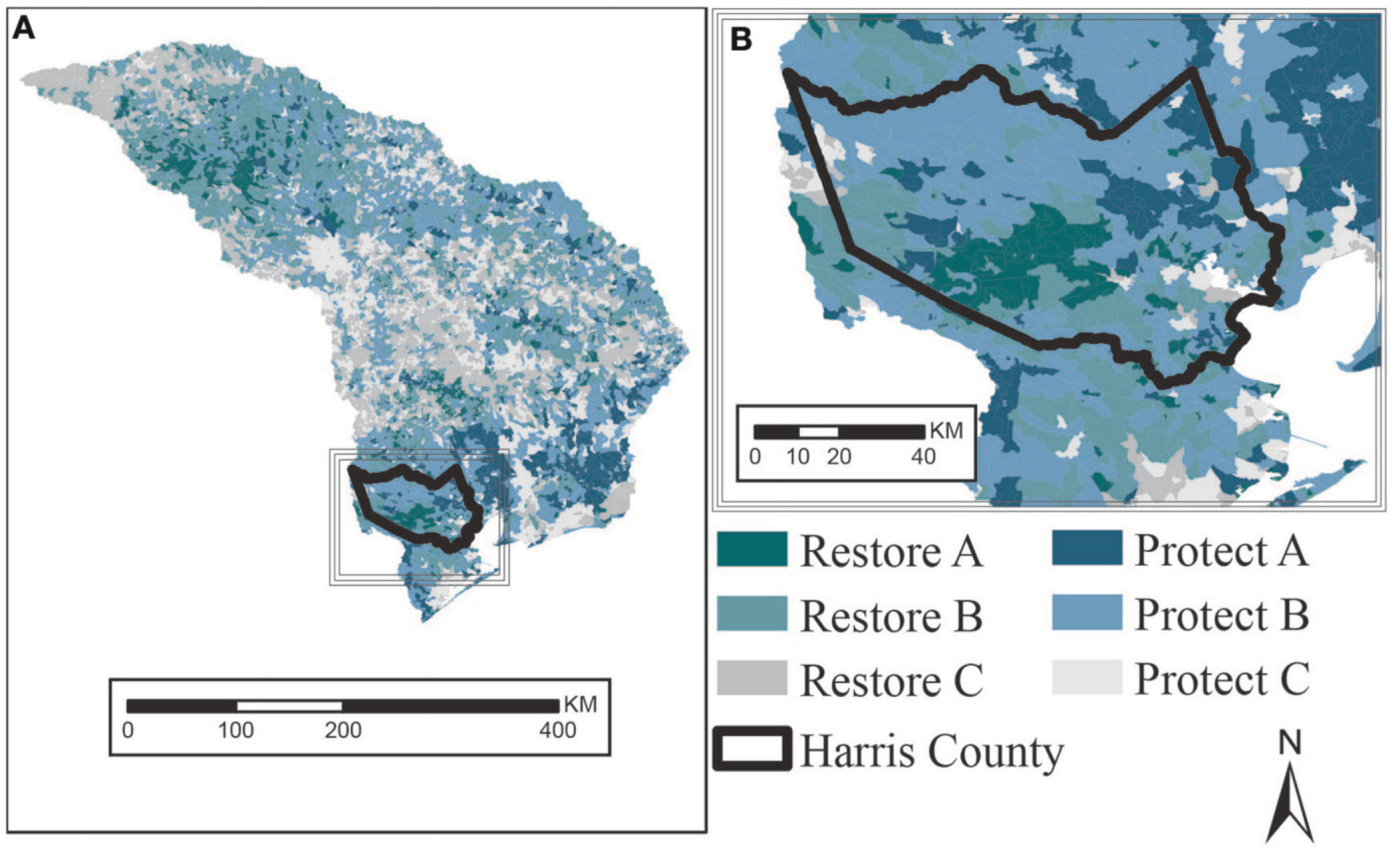
**(A)** Map of TX 12a sub-region catchments prioritized for conservation and restoration based on percent wetlands and flood-prone population within 4 km downstream (USEPA). **(B)** Map inset focused on outline of Harris County (FIPS 48201).

We combined catchment percent wetlands (Figure 7) and downstream population in flood-prone areas (Figure 9) as indicators of scarcity and demand (respectively) to perform a benefit assessment (Table 2) and map priority areas for management actions (Figure 10).

We labeled each catchment with a management action of either restore or protect based on scarcity, using the quartile percent of wetlands in the catchment (Table 2). We characterized catchments with a high percentage of wetlands (>50th percentile) as already benefitting people in flood-prone areas downstream and as candidates for protection. Likewise, we characterized catchments with a low percentage of wetlands (<50th percentile) as having the potential to benefit people in flood-prone areas downstream if the catchment wetlands increased through future restoration.

We assigned a priority of either A, B, or C based on demand, using the quartiles for downstream flood-prone populations (Table 2). We characterized the number of people in flood-prone areas downstream by quartile, where a higher quartile (>50th percentile) meant there are more people in flood-prone areas and demand for flood-reduction services is higher. Where demand for flood-reduction services is higher, we assumed restoration and conservation actions are higher priority (A or B) than where demand is low (<50th percentile is priority C).

Priority A catchments had to have both high demand (>75th quartile for downstream flood-prone population) and be either strong restoration candidates (<25th quartile for percent wetlands) or strong protection candidates (>75th quartile for percent wetlands). Priority B catchments had to have demand (>50th quartile for downstream flood-prone population) and were either restoration candidates (<50th quartile for percent wetlands) or protection candidates (>50th quartile for percent wetlands).

Priority C catchments were those with low demand (<50th downstream flood-prone population). A priority C catchment is only intended to indicate there are fewer people in flood-prone areas downstream making the wetlands in that catchment a lower priority than those that could benefit more people. This does not indicate that decision makers should neglect wetlands there. Since “Protect C” catchments may contain some limited wetlands (>50th quartile) it is possible there is a low downstream flood-prone population because of the flood-reduction benefits of these existing wetlands.

Mapping catchments by scarcity and demand indicator quartiles facilitated identification of priority areas for wetlands restoration or protection (Figure 10). A sub-region is too large for some decision makers to consider in its entirety, and decisions are often made within jurisdictional boundaries such as counties. There are 941 NHDPlus catchments that at least partially overlap Harris County. Figure 10 shows the location of Harris County within the sub-region to highlight how the scale of indicators allows for further analysis within a county. The highest restoration priorities cluster in an area southwest of Houston, whereas a cluster of protection priorities are to the northeast of the city.

## DISCUSSION

Datasets have previously characterized NHDPlusV2 catchments using census data (Hill et al., 2016). Those have been with the intended purpose of characterizing potential stressors on the landscape. Here the intended purpose was to define human beneficiaries for ecosystem services. The new dataset generated by this work was for this express purpose, and results show the utility of the dataset for characterizing a specific aspect of wetland restoration and conservation prioritization.

Characterizing NHDPlusV2 catchments with the two indicators, wetlands and population in flood-prone areas, allowed analysis and visualizations for decision-making at a variety of scales. The connection between catchments and the WBD allowed for aggregation to HUC-8, making it easier to see regional trends. The upstream and downstream relationships in the NHDPlusV2 allowed connections between wetlands, the flood-reduction services they provide, and the population in flood-prone areas downstream.

The two indicators we developed here may inform different decisions. The local scale example in Harris County, Texas showed how practitioners might prioritize restoration sites in the county to increase flood-reduction services where people need them most. The demonstration also showed how practitioners might prioritize existing freshwater wetlands for conservation to help ensure maintenance of important flood-reduction services. At this localized scale, we considered catchment data alongside other risk and socio-demographic indicators characterized for county and census-tract jurisdictional boundaries. The low natural infrastructure domain score in CRSI for Harris County identified the county as a good candidate for ecosystem restoration to increase resilience (Summers et al., 2017). Decision makers could use the indicators developed here to prioritize restoration projects to increase that domain score and increase resilience to flood events. Decision makers could also compare catchments identified as priorities for restoration or conservation in comparison to downstream demographics-based characterizations such as SoVI (Cutter et al.), to further differentiate specific catchments or projects as higher priority.

Actual restoration decisions should consider priority catchments identified here alongside additional characterizations of project feasibility and restorable wetland function. It is important to remember the limits of the prioritization method presented here. The method evaluates only two aspects, how many people may benefit and scarcity, of only one type of ecosystem service, flood-reduction. Our hope is that characterizing our metrics for NHDPlusV2 catchments and aggregating to HUC-8 will make it easier to integrate results with other datasets (e.g., EnviroAtlas; Pickard et al., 2015) or indicator frameworks (e.g., Recovery Potential Screening Tool; Norton et al., 2009) that provide additional information on wetland function and restoration feasibility at larger watershed scales.

In the example indicator application, we made several assumptions that may need adjustment depending on the context of the local decision. The first was the choice to use USEPA flood model results in lieu of FEMA flood zones. The demonstration here used the flood model results, because those are more comprehensive and therefore are more generally applicable. However, the FEMA flood zones are more specific to riverine flooding, better account for existing gray infrastructure, and should be more accurate where available. Characterizing the population in both these flood-prone areas allows future users to choose the flood definition that best suits their location and decision contexts. Both definitions for flooding rely on modeling and may not accurately represent where actual flood risk is highest (Batker et al., 2010; Yang et al., 2010).

The analysis here only considered the population in flood-prone areas, but uninhabited structures and infrastructure are exposed to flooding as well. The addition of these built environment assets may change restoration and conservation priorities. In their more localized RBI application, Mazzotta et al. (2018) used point datasets defining addresses for individual structures rather than population counts. Substituting actual flood occurrence data would be preferable where available.

The distance flood-reduction services flow downstream will impact where upstream freshwater wetland restoration and protection priorities are. In different parts of the United States and during different storms this distance will likely vary, and it is important to consider both local hydrologic characteristics and objectives. The brief sensitivity analysis performed here showed how changes in the downstream extent of flood reduction service flows may alter estimates of the number of people those services benefit, potentially altering priorities. However, the aggregation of datasets to NHDPlusV2 catchments with upstream and downstream relationships allows for these assumptions of downstream distances to be more easily and quickly manipulated.

The choice of method for binning combined indicators—in our case, based on sub-region quartiles—greatly influences the resulting prioritization. It is important to consider local conditions and, potentially, supplemental information when translating these metrics into indicators for prioritizing projects. For example, Harris County has a dense population compared to most of the Texas 12a sub-region, meaning a disproportionate number of catchments will be higher priority (i.e., have population in flood-prone areas) than in other less populated parts of the sub-region. Further, using a statistical distribution such as quartiles in prioritization does not account for potential thresholds. For example, in our Texas 12a sub-region demonstration the demand indicator (population in flood-prone areas) threshold between medium (Priority B) and low (Priority C) priorities was 5 people. In an actual decision, local context will dictate the required number of people serviced by a project for it to be a priority.

This analysis is very timely as Harris County voters recently approved 2.5 billion in bonds for flood risk reduction projects throughout the county and many of those proposed projects are nature-based, including wetlands restoration^3^. Benefit assessments can help inform decisions on where to prioritize for flood-reduction benefits and who might benefit from proposed projects. Decision makers can use national datasets such as the one generated here to make these benefit assessments more feasible and adapt them to fit the needs of a variety of different communities.

## Supplementary Material

Supplemental files

## Figures and Tables

**TABLE 1 |:** Fields characterizing NHDPlusV2 Catchments.

	Short name	Description	Field name
	FEATUREID	Unique catchment ID	COMID
Attributes	Percent wetlands	Percent of catchment in wetlands	wet_pct
	Flood-prone population (USEPA)	Dasymetric population in USEPA flood-prone areas	fldpop_EA
	Flood-prone population (FEMA)	Dasymetric population in FEMA flood-prone areas	fld_pop
Upstream/Downstream	Percent wetlands 4 km upstream	Percent of catchments within 4 km upstream in wetlands	wetpct_up
	Flood-prone population 4 km downstream (USEPA)	Dasymetric population in USEPA flood-prone areas for catchments within 4 km downstream	fldpopEAd
	Flood-prone population 4 km downstream (FEMA)	Dasymetric population in FEMA flood-prone areas for catchments within 4 km downstream	fld_popd

**TABLE 2 |:** Percent wetlands quartiles, flood-prone population downstream (USEPA, 4 km) quartiles, number of catchments and proportion of catchments for each decision category and priority.

Decision category	Catchment percent wetland (Quartiles)	Flood-prone population downstream (Quartiles)	Catchment count	Percent of catchments (%)
Restore A	<25th	>75th	1,367	5.6%
Restore B	<50th	>50th	4,383	17.8%
Restore C	<50th	<50th	6,554	26.6%
Protect A	>75th	>75th	1,672	6.8%
Protect B	>50th	>50th	4,881	19.8%
Protect C	>50th	<50th	5,750	23.4%

## Footnotes

1 The Oregon Explorer–Wetland Restoration Planning Tool, developed by Oregon State University and the Oregon Watershed Enhancement Board, can be accessed at: http://oe.oregonexplorer.info/wetlands/restoration/.

2 *Interactive Map of Mapped and Unmapped Areas.* FEMA Flood Map Service Center. Accessed at https://msc.fema.gov/portal/search.

3 Bond Program – Information and Community Engagement Interactive Map. Hosted by the Harris County Flood Control district. Accessed at: http://www.harriscountyfemt.org/cb.

## References

[R1] Acreman M, and Holden J. (2013). How wetlands affect floods. Wetlands 33, 773–786. doi: 10.1007/s13157-013-0473-2

[R2] Ahern J. (2007). “Green infrastructure for cities: the spatial dimension,” in Cities of the Future: Towards Integrated Sustainable Water and Landscape Management, eds. Novotny V. and Brown P. (London: IWA Publishing), 267–283.

[R3] ASFPM (2013). Flood Mapping for the Nation: A Cost Analysis for the Nation’s Flood Map Inventory. Madison, WI: Association of State Flood Plain Managers.

[R4] Bagstad KJ, Villa F, Batker D, Harrison-Cox J, Voigt B, and Johnson GW (2014). From theoretical to actual ecosystem services: mapping beneficiaries and spatial flows in ecosystem service assessments. Ecol. Soc 19:64. doi: 10.5751/ES-06523-190264

[R5] Batker D, Kocian M, Lovell B, and Harrison-Cox J. (2010). Flood Protection and Ecosystem Services in the Chehalis River Basin. Tacoma, WA: Earth Economics.

[R6] Bousquin J, Hychka K, and Mazzotta M. (2015). Benefit Indicators for Flood Regulation Services of Wetlands: A Modeling Approach. Washington, DC: US Environmental Protection Agency.

[R7] Bousquin J, Mazzotta M, and Berry W. (2017). Rapid Benefit Indicator (RBI) Spatial Analysis Toolset Manual. Washington, DC: US Environmental Protection Agency.

[R8] Brander LM, Brouwer R, and Wagtendonk A. (2013). Economic valuation of regulating services provided by wetlands in agricultural landscapes: a meta-analysis. Ecol. Eng 56, 89–96. doi: 10.1016/j.ecoleng.2012.12.104

[R9] Brander LM, Florax RJGM, and Vermaat JE (2006). The empirics of wetland valuation: a comprehensive summary and a meta-analysis of the literature. Environ. Resource Econ 33, 223–250. doi: 10.1007/s10640-005-3104-4

[R10] Brinson MM (1993). A Hydrogeomorphic Classification for Wetlands. Vicksburg, MS: US Army Corps of Engineers, 101.

[R11] Bullock A, and Acreman M. (2003). The role of wetlands in the hydrological cycle. Hydrol. Earth System Sci. Discuss 7, 358–389. doi: 10.5194/hess-7-358-2003

[R12] Burgess S. (2015). “12 Multifunctional green infrastructure: a typology,” in Handbook on Green Infrastructure: Planning, Design and Implementation, eds Sinnett D, Smith N, and Burgess S. (Cheltenham: Edward Elgar Publishing), 227.

[R13] Cutter SL, Boruff BJ, and Shirley W. (2003). Social vulnerability to environmental hazards. Soc. Sci. Q 84, 242–261. doi: 10.1111/1540-6237.8402002

[R14] Dahl TE (1990). Wetlands losses in the United States, 1780’s to 1980’s. Report to the Congress. No. PB-91–169284/XAB. St. Petersburg: FL National Wetlands Inventory.

[R15] Dahl TE, and Allord GJ (1996). “History of wetlands in the conterminous United States,” in National Summary on Wetland Resources. Reston, VA: USGS, Springfield, 19–26.

[R16] Doocy S, Daniels A, Murray S, and Kirsch TD (2013). The human impact of floods: a historical review of events 1980–2009 and systematic literature review. PLoS Curr. Disasters 16:1. doi: 10.1371/currents.dis.f4deb457904936b07c09daa98ee8171a PMC3644291 23857425

[R17] Environmental Law Institute (2012). Wetland and Stream Mitigation: A Handbook for Land Trusts. Washington, D.C: Environmental Law Institute.

[R18] FEMA (2017). National Flood Insurance Program Community Rating System Coordinator’s Manual. Washington, DC: Federal Emergency Management Agency, FIA-15/2017

[R19] FEMA (2018). FEMA Flood Map Service Center Portal. Website and web map. Available online at: https://msc.fema.gov/portal/search (accessed September 25, 2018).

[R20] Ghermandi A, Van Den Bergh JCJM, Brander LM, De Groot HLF, and Nunes PALD (2010). Values of natural and human-made wetlands: a meta-analysis. Water Resourc. Res 46:12. doi: 10.1029/2010WR009071

[R21] Handley L, and Wells C. (2009). Comparison of NLCD with NWI Classifications of Baldwin and Mobile Counties. Alabama: Geological Survey Report 2009–1058.

[R22] Hazards and Vulnerability Research Institute (2014). The Social Vulnerability Index, SoVI ®, Version 06–10. Columbia, SC: University of South Carolina. Available online at: http://www.sovius.org; https://coast.noaa.gov/digitalcoast/data/ (accessed September 27, 2018)

[R23] Healy M, and Secchi S. (2016). A comparative Analysis of Ecosystem Service Valuation Decision Support Tools for Wetland Restoration. Windham, NY: Association of State Wetland Managers.

[R24] Hey DL, and Philippi NS (1995). Flood reduction through wetland restoration: the Upper Mississippi River Basin as a case history. Restor. Ecol 3, 4–17. doi: 10.1111/j.1526-100X.1995.tb00070.x

[R25] Hill RA, Weber MH, Leibowitz SG, Olsen AR, and Thornbrugh DJ (2016). The Stream-Catchment (StreamCat) dataset: a database of watershed metrics for the Conterminous United States. J. Am. Water Resourc. Assoc 52, 120–128. doi: 10.1111/1752-1688.12372

[R26] Hollis GE (1975). The effect of urbanization on floods of different recurrence interval. Water Resour. Res 11, 431–435, doi: 10.1029/WR011i003p00431

[R27] Homer C, Dewitz J, Yang L, Jin S, Danielson P, Xian G, (2015). Completion of the 2011 national land cover database for the conterminous United States–representing a decade of land cover change information. Photogrammetr. Eng. Remote Sensing 81, 345–354.

[R28] Jongman B, Ward PJ, and Aerts JCJH (2012). Global exposure to river and coastal flooding: long term trends and changes. Global Environ. Change 22, 823–835. doi: 10.1016/j.gloenvcha.2012.07.004

[R29] Kadykalo AN, and Findlay CS (2016). The flow regulation services of wetlands. Ecosystem Services 20, 91–103. doi: 10.1016/j.ecoser.2016.06.005

[R30] Kelly NM (2001). Changes to the landscape pattern of coastal North Carolina wetlands under the Clean Water Act, 1984–1992. Landscape Ecol. 16, 3–16. doi: 10.1023/A:1008168322720

[R31] King DM, and Price EW (2004). Developing Defensible Wetland Mitigation Ratios. A Companion to “The Five-Step Wetland Mitigation Ratio Calculator,” Report prepared for NOAA Office of Habitat Conservation, Habitat Protection Division by King and Associates, Solomons Island, MD, 43.

[R32] King DM, Wainger LA, Bartoldus CC, and Wakeley JS (2000). Expanding Wetland Assessment Procedures: Linking Indices of Wetland Function with Services and Values. Wetlands Research Program, US Army Corps of Engineers.

[R33] Liu W, Chen W, and Peng C. (2014). Assessing the effectiveness of green infrastructures on urban flooding reduction: A community scale study. Ecol. Model 291, 6–14. doi: 10.1016/j.ecolmodel.2014.07.012

[R34] Mazzotta M, Bousquin J, Berry W, Ojo C, McKinney R, Hychka K, (2018). Evaluating the ecosystem services and benefits of wetland restoration using the rapid benefit indicators approach. Integr. Environ. Assess. Manage doi: 10.1002/ieam.4101 PMC6475909 30246394

[R35] Mazzotta M, Bousquin J, Ojo C, Hychka K, Gottschalk Druschke C, Berry W, (2016). Assessing the Benefits of Wetland Restoration: A Rapid Benefit Indicators Approach for Decision Makers. Narragansett, RI: USEPA, Office of Research and Development. EPA/600/R-16/084.

[R36] McKay L, Bondelid T, Dewald T, Johnston J, Moore R, and Rea A. (2012). NHDPlus Version 2: User Guide. Washington, DC: National Operational Hydrologic Remote Sensing Center.

[R37] Merz B, Kreibich H, Schwarze R, and Thieken A. (2010). Assessment of economic flood damage. Nat. Hazards Earth Syst. Sci 10, 1697–1724. doi: 10.5194/nhess-10-1697-2010

[R38] Miller N, Kline J, Bernthal T, Wagner J, Smith C, Axler M, (2017). Wetlands by Design: A Watershed Approach for Wisconsin. Madison, WI: Wisconsin Department of Natural Resources and The Nature Conservancy. Available online at: http://maps.freshwaternetwork.org/wisconsin/ (accessed September 26, 2018).

[R39] Miller NA, and Golet FC (2001). Development of a Statewide Freshwater Wetland Restoration Strategy. Final Research Report prepared for RI DEM Office of Water Resources and U.S. EPA Region 1. Kingston, RI: University of Rhode Island.

[R40] Milly PCD, Betancourt J, Falkenmark M, Hirsch RM, Kundzewicz ZW, Lettenmaier DP, (2008). Stationarity is dead: whither water management? Science 319, 573–574. doi: 10.1126/science.1151915 18239110

[R41] Mitsch WJ, and Gosselink JG (2000). The value of wetlands: importance of scale and landscape setting. Ecol. Econ 35, 25–33. doi: 10.1016/S0921-8009(00)00165-8

[R42] Nelson E, Mendoza G, Regetz J, Polasky S, Tallis H, Cameron D, (2009). Modeling multiple ecosystem services, biodiversity conservation, commodity production, and tradeoffs at landscape scales. Front. Ecol. Environ 7, 4–11. doi: 10.1890/080023

[R43] Norton DJ, Wickham JD, Wade TG, Kunert K, Thomas JV, and Zeph P. (2009). A method for comparative analysis of recovery potential in impaired waters restoration planning. Environ. Manage 44, 356–368. doi: 10.1007/s00267-009-9304-x 19452204

[R44] Pickard BR, Daniel J, Mehaffey M, Jackson LE, and Neale A. (2015). EnviroAtlas: a new geospatial tool to foster ecosystem services science and resource management. Ecosyst. Services 14, 45–55. doi: 10.1016/j.ecoser.2015.04.005

[R45] Samuels WB, Springer JC, and Fry R. (2014). An Effective Tool for Drinking Water Protection ESRI. Available online at: http://www.esri.com/$\sim$/media/Files/Pdfs/library/?iers/pdfs/drinking-water-protection.pdf (accessed October, 2018).

[R46] Shuster WD, Bonta J, Thurston H, Warnemuende E, and Smith DR (2005). Impacts of impervious surface on watershed hydrology: a review. Urban Water J. 2, 263–275. doi: 10.1080/15730620500386529

[R47] Stein ED, and Ambrose RF (1998). Cumulative impacts of Section 404 Clean Water Act permitting on the riparian habitat of the Santa Margarita, California Watershed. Wetlands, 18, 393–408. doi: 10.1007/BF03161533

[R48] Summers JK, Harwell LC, Buck KD, Smith LM, Vivian DN, Bousquin JJ, (2017). Development of a Climate Resilience Screening Index (CRSI): An Assessment of Resilience to Acute Meteorological Events and Selected Natural Hazards. Gulf Breeze, FL: USEPA, Office of Research and Development. EPA600/R-17/238.

[R49] Summers JK, Harwell LC, Smith LM, and Buck KD (2018). Measuring community resilience to natural hazards: the Natural Hazard Resilience Screening Index (NaHRSI)–Development and Application to the United States. GeoHealth 2, 372–394. doi: 10.1029/2018GH000160 32159008 PMC7007161

[R50] USDA (2015). Conservation Reserve Program – Floodplain Wetlands Initiative Brief. United States Department of Agriculture, Farm Service Agency, (accessed Septmeber 24, 2018).

[R51] USEPA (2015). EnviroAtlas – Dasymetric Allocation of Population Fact Sheet. April 2015. Washington, DC: Environmental Protection Agency. Available online at: https://enviroatlas.epa.gov/enviroatlas/DataFactSheets/pdf/Supplemental/DasymetricAllocationofPopulation.pdf (accessed september 25, 2018).

[R52] USEPA (2016). Dasymetric Allocation of Population, Raster Dataset. Washington, DC: Environmental Protection Agency. Available online at: http://newftp.epa.gov/epadatacommons/ORD/EnviroAtlas/dasymetric_us_20160208.zip (accessed Septmeber 25, 2018).

[R53] USFW (2018). National Wetlands Inventory Dataset. Washington, DC: US department of the Interior, Fish and Wildlife Service. Availble online at: https://www.fws.gov/wetlands/Data/State-Downloads.html (accessed Septmeber 25, 18).

[R54] VanSickle J, and Burch-Johnson C. (2008). Parametric distance weighting of landscape influence on streams. Landscape Ecol. 23, 427–438. doi: 10.1007/s10980-008-9200-4

[R55] Wing OE, Bates PD, Smith AM, Sampson CC, Johnson KA, Fargione J, (2018). Estimates of present and future flood risk in the conterminous United States. Environ. Res. Lett 13:034023. doi: 10.1088/1748-9326/aaac65 PMC11977400 40201223

[R56] Woznicki SA, Baynes J, Panlasigui S, Mehaffey M, and Neale A. (2019). Development of a spatially complete floodplain map of the conterminous United States using random forest. Sci. Total Environ 647, 942–953. doi: 10.1016/j.scitotenv.2018.07.353 30180369 PMC8369336

[R57] Yang W, Wang X, Liu Y, Gabor S, Boychuk L, and Badiou P. (2010). Simulated environmental effects of wetland restoration scenarios in a typical Canadian prairie watershed. Wetlands Ecol. Manag 18, 269–79. doi: 10.1007/s11273-009-9168-0

