# Supplementary material for: A Geospatial Assessment of Flood Vulnerability Reduction by Freshwater Wetlands–A Benefit Indicators Approach
Source: Front Environ Sci. Author manuscript; Available in PMC 2021 Jul 26. (PMC8312689; doi:10.3389/fenvs.2019.00054)
Supplement: Supplemental files [file NIHMS1530227-supplement-Supplemental_files.zip › Table_1_A Geospatial Assessment of Flood Vulnerability Reduction by Freshwater Wetlands–A Benefit Indicators Approach.docx]

Supplementary Material

**A Geospatial Assessment of Flood Vulnerability Reduction by Freshwater Wetlands – A Benefit Indicators Approach**

Justin Bousquin*, and Kristen Hychka

*** Correspondence:** Justin Bousquin: bousquin.justin@epa.gov

# Supplementary Materials

This document details additional processing steps, the comparison of results to existing datasets, the sensitivity of results to the distance downstream where we considered flooding, catchment metrics included in supplementary table not detailed in the main text, and examination of results from a second perspective, looking upstream. In addition to this document there are two other types of supplementary materials:

1. A compressed file (.zip) of the Python scripts (.py) used to download NWI datasets, to characterize catchments metrics using datasets, and to aggregate catchment metrics upstream/downstream.
2. Compressed files (.zip) of comma separated values (.csv) containing catchment metrics for each of the 19 HUC-2 regions.

# Data Processing

NHDPlusV2 distributes catchments geographies and catchment attribute tables in sections divided into 21 Vector Processing Units (VPUs) and subdivided into 59 Raster Processing Units (RPUs). RPUs are referred to as “sub-regions” in the main text. We split input datasets and catchments into sections for processing based on the same sub-regional basins.

NHDPlusV2 integrated the latest Watershed Boundary Dataset (WBD). Integration with WBD means that the first 8 digits of the NHD river reach codes match up to an eight-digit HUC-8. These reach codes were used to aggregate catchment metrics up to HUCs. To aggregate catchment percent-area based characterizations (e.g. wetlands percent), scripts back calculated the original feature area using the original catchment area and then totaled the wetland area across the combined catchments. This additional step helped to ensure the aggregate percentage was representative of the entire area it summarized without bias to smaller catchments.

# Comparison to Existing Datasets

Both the NWI wetland and the dasymetric population catchment datasets have similar datasets already aggregated to catchments within StreamCat (Hill et al. 2016). StreamCat captures the percent of catchment area in wetlands based on wetland cover classes in the National Land Cover Database (NLCD; Homer et al. 2015). NLCD classifies wetlands as either woody or herbaceous but does not distinguish between coastal and freshwater wetlands. Although both wetland types provide flood-reduction benefits, the generation and flow of those benefits are different. Upland freshwater wetlands attenuate flood water that would otherwise contribute to flooding downstream. This contrasts coastal wetlands that reduce flooding by reducing storm surge energy, acting as a barrier to water coming inland from the coast. We combined the NLCD defined “herbaceous wetlands” (PctHbWet2011Cat field) and “woody wetlands” (PctWdWet2011Cat field) to compare to percent NWI wetlands. We multiplied population density (PopDen2010Cat field) by catchment area to get the total catchment population. We compared these two catchment values for wetlands and population to our catchment results on a national scale (**Supplementary Figure 1**).

NWI wetlands matched NLCD wetlands very well. Areas that had higher catchment percentages from the NWI dataset appear in red in Figure 1, whereas areas that had higher catchment percentages from the NLCD based dataset appear in blue. Higher differences in the two datasets appear mainly in areas where catchment wetland percentages are higher; the great lakes region, south-east coast and areas along the gulf coast. Some of these differences may be due to the exclusion of certain types of wetlands from the NWI definitions (e.g. coastal wetlands). Some differences may be due to the resolution of the datasets defining wetlands. The NLCD definitions are raster based, meaning every 900m^2^ (30m x 30m cell) either was or was not wetlands. The NWI definitions are polygon based, meaning smaller portions of each catchment were differentiated as wetlands or not wetlands.

StreamCat (Hill et al. 2016) used block-level population counts from the 2010 census to characterize catchment population. In this tabular, each catchment has a total population and a consistent population density across the catchment would have to be estimated, then multiplied by the flood-prone area. This method would likely over-estimate the population in flood-prone areas since it assumes the same population density in known waterbodies. Another option was to overlay the original block-level population counts from the 2010 census with flood-prone areas. Although this method would account for differences in block-population densities within each catchment, census block geographies still overlap known waterbodies. Dasymetric maps attempt to redistribute the population within a census block based on ancillary data. The EnviroAtlas provides a dasymetric map from the same 2010 census block data used by StreamCat for previous estimates. We used this population map to characterize catchment flood-prone population. We characterized catchments using population from the dasymetric map before limiting it to flood-prone areas to be able to compare it directly to StreamCat estimates (**Supplementary** **Figure 1**). Catchments in Figure 1 appear in red when the dasymetric population was higher, and blue when the census block based population was higher. Although there were many small differences in total population, these were typically nearby catchments that had similar differences in the opposite direction.


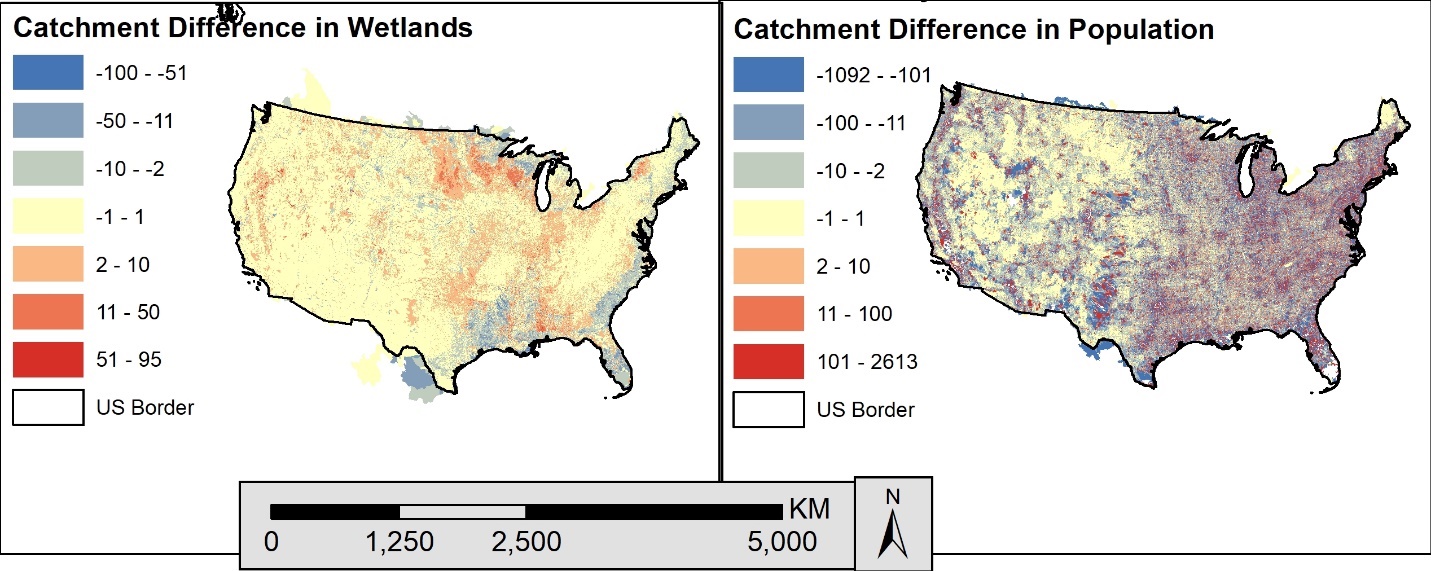


**Supplementary Figure 1.** Data comparison maps. Catchment differences in wetlands percentage (left) were calculated by subtracting, wet_pct, the NWI based catchment percentage from the total of herbaceous wetlands, PctHbWet2011Cat, and woody wetlands, PctWdWet2011Cat. Catchment differences in population (right) were calculated by subtracting the dasymetric population from the census-based estimate, PopDen2010Cat, after converting the latter from a population density to a total population.

# Sensitivity Analysis

Understanding the sensitivity of results to the upstream or downstream threshold distance is vital. The distance flood reduction benefits travel downstream may be different for different areas, different wetlands, or even for the same wetland during different storms (Bousquin et al. 2015; Liu et al. 2014; Mitsch and Gosselink 2000; Van Sickle and Burch Johnson 2008). We aggregated downstream population in flood prone areas using three different distance thresholds, 4 km, 5 km, and 8 km. Bousquin et al. (2015) performed simulations that showed 4-km was the median downstream distance where flood reductions dissipated to undetectable levels when adding upstream wetlands. The median distance in simulations shifted to flooding 8-km downstream only when upstream wetland additions were extensive or during extreme storm events. Although many small increases in the flood prone population were expected, our interest was in how these changes might impact catchment categorizations using our quartile method and how changes in catchment categorization might alter decisions that were based on the characterizations.

Altering the downstream distance threshold for flood-prone population resulted in minor changes in the quartile definitions for the TX 12a sub-regional basin (**Supplementary Table 1**). Only the 3^rd^ and 4^th^ quartiles changed when 5 km downstream was considered in place of 4 km, the 3^rd^ quartile widening from 5-16 people to 5-20 people. All four quartiles changed when 8 km downstream was considered in place of 4 km, but again only marginally.

**Supplementary Table 1**. Quartile thresholds for flood-prone population at different downstream distances thresholds. Table shows thresholds for TX 12a sub-regional basin.

|  | Quartile upper threshold | | |
| --- | --- | --- | --- |
| Quartiles | 4 km | 5 km | 8 km |
| <25^th^ | 1 | 1 | 2 |
| 25^th^-50^th^ | 5 | 5 | 8 |
| 50^th^-75^th^ | 16 | 20 | 31 |
| 75^th^-100^th^ | 25,104 | 25,104 | 25,104 |

When the distance downstream considered changed from 4km to 8km, it resulted in the quartile category for people in flood-prone areas changing for 35% of catchments (n=8495; **Supplementary** **Table 2**). However, a change in catchment flood quartile may not alter the priority level for increased flood mitigation upstream if it remains in the bottom two quartiles, as was the case for 11% of the catchments (n=2740). Priority was reduced in 12.8% (n = 3151), while priority increased in 10.6% (n=2604), either to B (5.9%; n=1441), or A (4.7%; n=1163).

**Supplementary Table 2**. Priority changes when flood-prone population downstream distance threshold was changed from 4 km to 8 km. Table shows thresholds for TX 12a sub-regional basin.

| Flood Quartile Change | Catchment Count | Priority Change |
| --- | --- | --- |
| 0.00 -> 0.75 | 244 | C to A |
| 0.25 -> 0.75 | 303 | C to A |
| 0.50 -> 0.75 | 616 | B to A |
| 0.00 -> 0.50 | 556 | C to B |
| 0.25 -> 0.50 | 885 | C to B |
|  |  |  |
| 0.75 -> 0.50 | 1163 | A to B |
| 0.50 -> 0.25 | 1988 | B to C |
|  |  |  |
| 0.00 -> 0.25 | 970 | No Change |
| 0.25 -> 0.00 | 1770 | No Change |

No strong spatial trends emerged because of changes in decision category due to changes in the downstream distance considered (**Supplementary** **Figure 2**). One potential trend that may merit more investigation is that in highly populated areas, like Dallas and Houston, there seem to be fewer changes in decision category.

**
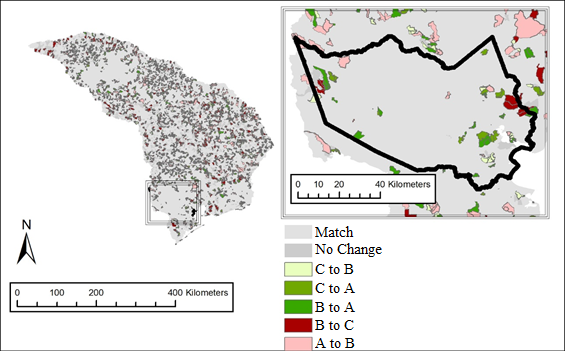
**

**Supplementary Figure 2.** Map of changes in catchment action where downstream distance considered for flood prone population was changed from 4-km to 8-km within the TX 12a sub-regional basin.

# Additional Fields in Supplementary Data Tables

Input datasets stopped at international borders or contained missing data in some locations. We estimated the percent of each catchment that was missing values for each raster input dataset. Each catchment metric derived from a raster dataset has an additional field characterizing percent missing. This value allows users to evaluate the appropriateness of the data for their analysis (**Supplementary** **Table 3**). Vector input datasets (e.g. NWI) stop at international borders and may contain missing values in some locations as well. However, vector datasets do not identify areas of missing values and catchments do not have percent missing for these data. For FEMA flood zones, we encourage users to evaluate missing values using FIRM boundaries^[[1]](#footnote-1)^.

**Supplementary Table 3**. Fields characterizing NHDPlusV2 Catchments.

|  | **Field Name** | **Alias** | **Description** |
| --- | --- | --- | --- |
|  | COMID | FEATUREID | Unique catchment ID |
| Attributes | wet_pct | Percent wetlands | Percent of catchment in wetlands |
|  | fldpop_EA | Flood-prone population | Dasymetric population in USEPA flood-prone areas |
|  | fld_pop | Flood-prone population | Dasymetric population in FEMA flood-prone areas |
|  | dasy_pop | Total population | Dasymetric population in catchment |
|  | dasypopNA | Percent NA | Percent of catchment missing dasymetric population |
|  | fldpct_EA | Percent flood-prone | Percent of catchment in EnviroAtlas flood-prone area |
|  | fldpct_NA | Percent NA | Percent of catchment missing EnviroAtlas flood-prone area |
|  | fld_pct | Percent flood-prone | Percent of catchment in FEMA flood-prone area |
| Upstream/Downstream | wetpct_up | Percent wetlands 4km upstream | Percent of catchments within 4 km upstream in wetlands |
|  | fldpopEAd | Flood-prone population 4km downstream | Dasymetric population in USEPA flood-prone areas for catchments within 4 km downstream |
|  | fld_popd | Flood-prone population 4km downstream | Dasymetric population in FEMA flood-prone areas for catchments within 4 km downstream |
|  | dasy_popd | Total population 4km downstream | Dasymetric population in catchments within 4 km downstream |
|  | EA_pct_d | Flood-prone area 4km downstream | Percent of catchments within 4 km downstream in EnviroAtlas flood-prone area |
|  | fld_pct_d | Flood-prone area 4km downstream | Percent of catchments within 4 km downstream in FEMA flood-prone area |

# Looking Upstream

In the main text, the perspective taken was that of a practitioner looking for catchments to focus wetland restoration or protection efforts in. The second perspective, presented here, is that of potential beneficiaries looking for areas upstream where restoration would help them specifically. From that perspective the indicators are (1) local beneficiaries, i.e. how many people are in flood prone areas, and (2) upstream scarcity, i.e. how many upstream wetlands are already supplying flood-reduction services.

We made a second catchment characterization from the same datasets by taking the perspective of the beneficiary, based on catchment flood-prone population, and looking upstream to estimate wetlands that may be providing those beneficiaries with reduced flooding. Figure 3 shows a similar map of prioritization and restoration using the same quartile approach. The main difference is in perspective, as this map shows the catchments where people who would most benefit from protection or restoration of upstream wetlands are present. People in flood-prone areas within these catchments are in a position to benefit from the actions upstream. These maps help identify stakeholders that may benefit from wetland restoration or protection. This may draw attention to projects outside of municipal boundaries that would be beneficial for people living within those boundaries. These maps can also integrate other demographic characterizations, such as the social vulnerability index (Cutter et al. 2003), which can help differentiate specific catchments or projects as even higher priority.

**
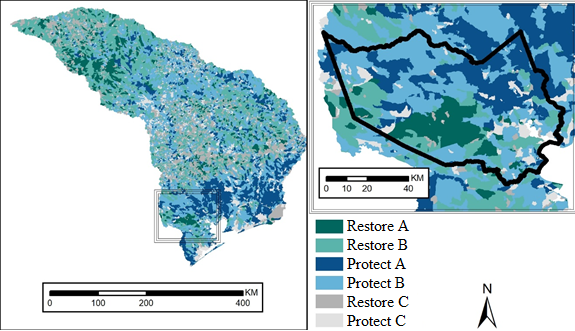
**

**Supplementary Figure 3.** Maps highlighting catchments with populations that could benefit from conservation and restoration upstream. (left) Map of TX 12a regional sub-basin catchments prioritized for upstream conservation and restoration based on percent catchment in NWI wetlands upstream and flood populations, based on EnviroAtlas. (right) Map inset focused on outline of Harris County (FIPS 48201).

**References**

Bousquin, J., Hychka, K., and Mazzotta, M. (2015). Benefit indicators for flood regulation services of wetlands: a modeling approach. US Environmental Protection Agency, Washington, DC. EPA/600/R-15/191

Cutter, S. L., Boruff, B.J., and Shirley, W. (2003). Social vulnerability to environmental hazards. *Soc. Sci. Q.* 84, 242-261. doi: 10.1111/1540-6237.8402002

Hill, R.A., Weber, M.H., Leibowitz, S.G., Olsen, A.R., and Thornbrugh, D.J., (2016). The Stream‐Catchment (StreamCat) Dataset: A Database of Watershed Metrics for the Conterminous United States. *J. Am. Water Resour. Assoc.* 52(1): 120‐128. doi: 10.1111/1752‐1688.12372

Homer, C., Dewitz, J., Yang, L., Jin, S., Danielson, P., Xian, G., et al. (2015). Completion of the 2011 national land cover database for the conterminous United States-representing a decade of land cover change information. *Photogrammetr. Eng. Remote Sensing* 81, 345-354.

Liu, W., Chen, W., and Peng, C. (2014). Assessing the effectiveness of green infrastructures on urban flooding reduction: A community scale study. *Ecological Modelling*, 291, 6-14. doi: 10.1016/j.ecolmodel.2014.07.012

Mitsch, W.J. and Gosselink, J.G. (2000). The value of wetlands: importance of scale and landscape setting. *Ecological Economics*, 35(1), 25-33. doi: 10.1016/S0921-8009(00)00165-8.

VanSickle, J., and Burch-Johnson, C. (2008). Parametric distance weighting of landscape influence on streams. *Landscape Ecology*, 23(4), 427-438. doi: 10.1007/s10980-008-9200-4.

1. Interactive map of mapped and unmapped areas. FEMA Flood Map Service Center. Accessed at https://msc.fema.gov/portal/search [↑](#footnote-ref-1)
